# Psychosis as the Initial Presenting Symptom of Anti-Hu Encephalitis: A Case Series with Literature Review

**DOI:** 10.1192/j.eurpsy.2022.1690

**Published:** 2022-09-01

**Authors:** E. Garrels, A. Zamiri, S. Pakniyat-Jahromi, S. Gunturu

**Affiliations:** BronxCare Health System, Psychiatry, Bronx, United States of America

**Keywords:** Psychosis, Anti-Hu, Encephalitis

## Abstract

**Introduction:**

Anti-Hu related Paraneoplastic Neurological Syndrome (PNS) is one of the most common paraneoplastic-associated neurological syndromes (Kayser 2010). While the primary clinical manifestations are neurologic in nature (Smitt 2002), only rare reports exist regarding psychiatric manifestations. Our poster presents two cases of Anti-Hu Encephalitis manifesting as psychosis as well as a systematic literature review on the co-occurrence of psychosis and PNS.

**Objectives:**

The aim of this case series is to show psychosis as the primary symptom of a paraneoplastic syndrome that does not typically present in this way. It also serves as a reminder to have a detailed work-up and maintain a wide differential diagnosis when evaluating patients with first-episode psychosis.

**Methods:**

Two cases of anti-Hu encephalitis primarily presenting with psychiatric symptoms are discussed. A systematic literature review was carried out based on the Preferred Reporting Items for Systematic Reviews and Meta-Analyses (PRISMA) model on three electronic databases: PubMed, Embase, and PsycINFO. Search terms included were (Anti-Hu) AND (Psychosis OR Hallucinations OR Schizophrenia OR Schizoaffective).

**Results:**

Our case series reports on two patients with diagnosed anti-Hu encephalitis who were treated by our psychiatry team, where the primary manifestations of the illness were psychiatric in nature. Psychotic symptoms in these cases were managed with Risperidone, Olanzapine, and Paliperidone.

**Conclusions:**

Psychotic symptoms are seldom reported in the literature and cases like the ones presented emphasize the importance of a full medical work-up for first episode psychosis as well as a wide differential. Given the increased association between PNS and psychiatric illness, more emphasis and further research is warranted.

**Disclosure:**

No significant relationships.

